# Fish Mercury and Surface Water Sulfate Relationships in the Everglades Protection Area

**DOI:** 10.1007/s00267-013-0224-4

**Published:** 2014-01-03

**Authors:** Mark C. Gabriel, Nicole Howard, Todd Z. Osborne

**Affiliations:** 1 USEPA/Office of Research and Development (ORD)/National Exposure Research Laboratory (NERL)/Ecosystem Research Division (ERD), 960 College Station Rd., Athens, GA 30605 USA; 2 Wetland Biogeochemistry Laboratory, Soil and Water Science Department, University of Florida, 2181 McCart Hall, P.O. Box 11029, Gainesville, FL 32611 USA; 3 The Whitney Laboratory for Marine Bioscience, 9505 Ocean Shore Blvd., St. Augustine, FL 320808 USA

**Keywords:** Mercury, Sulfate, Everglades, Bioaccumulation, CERP, Fish

## Abstract

Few published studies present data on relationships between fish mercury and surface or pore water sulfate concentrations, particularly on an ecosystem-wide basis. Resource managers can use these relationships to identify the sulfate conditions that contain fish with health-concerning total mercury (THg) levels and to evaluate the role of sulfate in methyl-mercury (MeHg) production. In this study, we derived relationships between THg in three fish trophic levels (mosquitofish, sunfish, and age-1 largemouth bass) and surface water sulfate from 1998 to 2009 for multiple stations across the Everglades Protection Area (EPA). Results show the relationship between sulfate and fish THg in each fish type is nonlinear and largely skewed, similar to the relationship between MeHg production and sulfate concentration in peatland sediment pore water identified by other researchers. Peak fish THg levels occurred in ~1 to 12 mg/L sulfate conditions. There was significant variability in the fish THg data, and there were several instances of high-fish THg levels in high-sulfate conditions (>30 mg/L). Health-concerning fish THg levels were present in all surface water sulfate conditions; however, most of these levels occurred in 1–20 mg/L sulfate. The data in this study, including recent studies, show consistent and identifiable areas of high- and low-fish THg across the spectrum of surface water sulfate concentration, therefore, applying an ecosystem-wide sulfur strategy may be an effective management approach as it would significantly reduce MeHg risk in the EPA.

## Introduction

Over the recent 20 years, the South Florida ecosystem has experienced excessive mercury bioaccumulation, resulting in widespread fish consumption advisories (FDOH 2008). High levels of mercury in biota within the greater Everglades were first reported by Ogden et al. (1974). During the same period, Andren and Harriss (1973) observed enhanced methyl-mercury (MeHg) production in sediments. In 1988, reports of mercury levels exceeding 1 mg/kg in largemouth bass (LMB) (*Micropterus salmoides*) in South Florida Water Conservation Areas (WCAs) prompted intensive mercury monitoring and assessment in fish and wildlife by state and federal agencies.

The primary concern with mercury in ecosystems is the production and bioaccumulation of MeHg, a neurotoxin that poses a threat to humans (USEPA 2013) and wildlife (Eisler 1987; Spalding et al. 2000) who consume fish and other biota. High levels of MeHg have been linked to impaired reproduction and survival, developmental, and behavioral abnormalities and mortality. Field and laboratory experiments demonstrate that production of MeHg is enhanced by microbial sulfate reduction (MSR) under anoxic conditions (Gilmour et al. 1992, 1998; Benoit et al. 2003; Harmon et al. 2004, 2007; Jeremiason et al. 2006; Mitchell and Branfireun 2008; Shao et al. 2012). In the process of using oxidized inorganic sulfur (sulfate [SO_4_^2−^]) for energy purposes, particular strains of sulfate reducing bacteria (Gilmour et al. 2011) methylate bioavailable inorganic oxidized mercury (Hg^2+^) to MeHg (largely as mono-methyl [CH_3_Hg^+^]) which efficiently bioaccumulates in the food chain. Certain species of mercury, namely small neutrally charged mercury complexes (e.g., HgS^0^), enter sulfate-reducing bacteria through passive diffusion (Benoit et al. 1999a; Jay et al. 2002; Gilmour et al. 2011). Given that, sulfate is a primary driver for MSR; a limiting condition is the concentration of sulfate in surface and pore water. Persistent MeHg production in the greater Everglades is primarily a result of a large quantity of (1) bioavailable mercury delivered by atmospheric deposition, (2) electron donors [labile dissolved organic carbon (DOC), e.g., lactate, acetate] (Achá et al. 2011), and (3) electron acceptors—in this case sulfate (Orem et al. 2011). Other Everglades factors such as circumneutral pH and high-water temperature also aid efficient development of MeHg.

Due to the large impact of MSR on MeHg production, a valuable analysis is determining relationships between mercury in biota and sulfate. There has been significant investigation into the impact of sulfate on mercury methylation through the examination of MSR activity (Gilmour et al. 1992, 2007; King et al. 1999; Marvin-DiPasquale et al. 2003; Jeremiason et al. 2006); however, few studies have presented data on the relationship between sulfate and fish THg, particularly on an ecosystem-wide basis. This rather simple derivation can be useful for resource managers to understand sulfate/fish THg dynamics and provide guidance in the protection of sensitive ecosystems against excessive mercury bioaccumulation. Specifically, these relationships may be used by resource managers to identify the sulfate conditions that contain fish with health-concerning THg levels. Dissolved sulfate does not have a direct influence on facilitating MeHg bioaccumulation through the food chain in the Everglades ecosystem (there may be indirect effects through fish physiology and growth), but as previously documented, sulfate has an important role in MeHg production. MeHg bioaccumulation is primarily a function of fish and aquatic biota feeding patterns, diet and migration (Simoneau et al. 2005; Eagles-Smith et al. 2008; Baker et al. 2009; Li et al. 2009; Riva-Murry et al. 2011). As a result, the relationship between sulfate and fish mercury may be unclear or nonexistent using data on a site-by-site basis; however, relationships may be revealed with large data sets that span an ecosystem. From human and wildlife health perspectives, the scientific community and government agencies are mainly concerned with fish THg levels; therefore, this evaluation provides an interpretation of sulfate’s role as a potential tool for management response. Accordingly, the objectives of this study were to: (1) evaluate relationships between mercury concentration in three fish trophic levels (mosquitofish, sunfish, and LMB) and surface water sulfate using 11 years (1998–2009) of data across the Everglades Protection Area (EPA), and (2) determine what these relationships imply for Everglades management and restoration.

## Materials and Methods

### Monitoring Stations

**Fig. 1:**
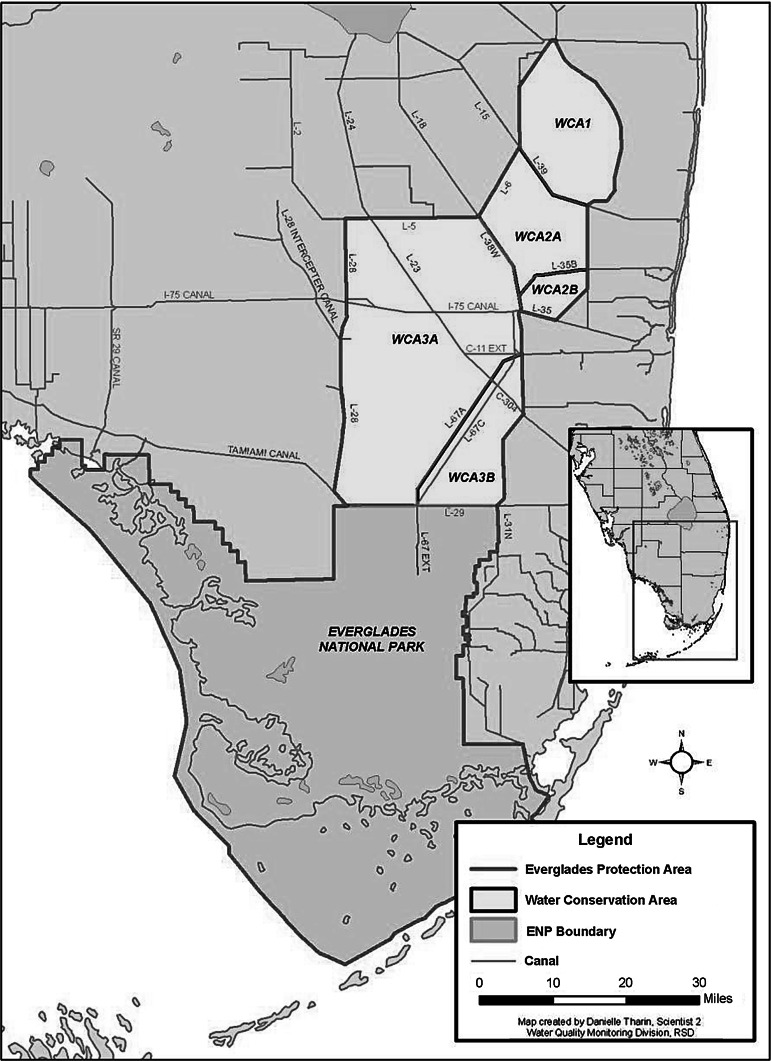
The Everglades Protection Area consists of Water Conservation Areas *1*, *2A*, *2B*, *3A* and *3B*, and Everglades National Park

**Fig. 2:**
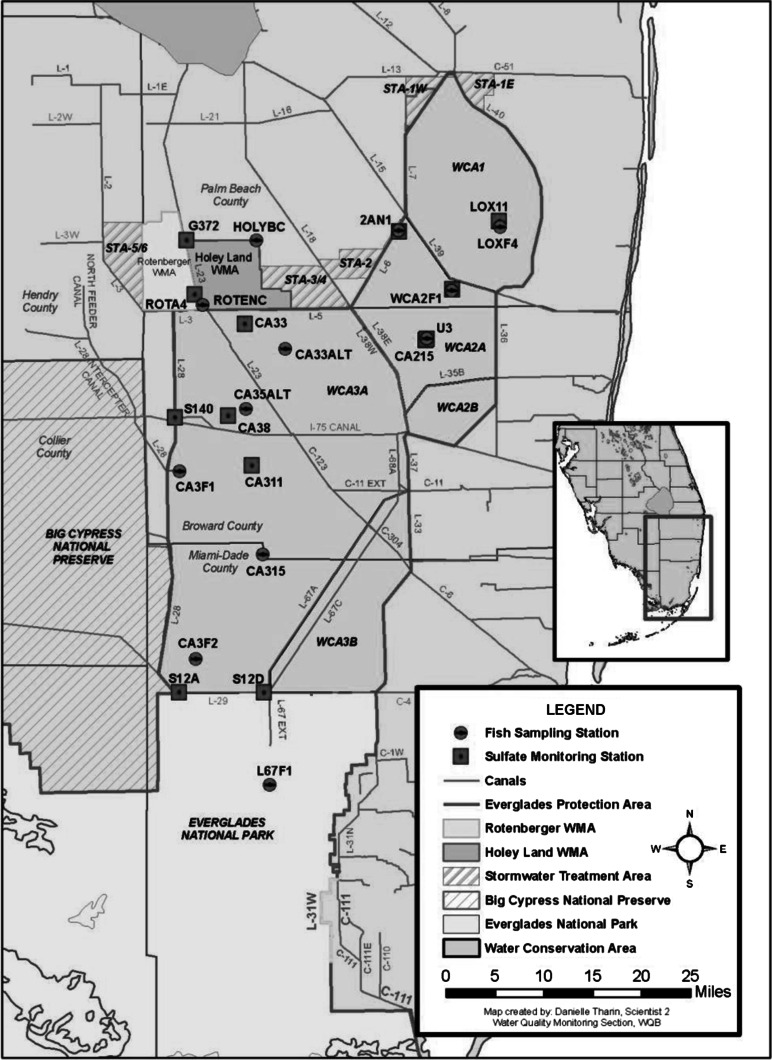
Fish THg and sulfate collection site locations in 2009

The data used in the study were obtained from the South Florida Water Management District’s (SFWMD) DBHydro Database (http://www.sfwmd.gov/dbhydroplsql/show_dbkey_info.main_menu: accessed June, 2010). As a condition of its operating permits and the 1991 Settlement Agreement for the US versus SFWMD; Florida Department of Environmental Regulations, Case No. 88-1886-CIV-HOEVELER, SFWMD is required to monitor mercury in fish tissue and sulfate in surface water at various locations throughout the EPA (Fig. 1). The stations used in this study (Fig. 2) cover a relatively wide range of biogeochemical conditions including sulfate and mercury levels within the EPA which negates any biased assessment of mercury methylation potential. The fish- and sulfate-monitoring stations were matched based on their proximity and hydrologic connection to each other over the analysis period of 1998–2009. Locations and names of these stations have varied over time due to changes in hydrology, land access, and mandated monitoring requirements. The stations presented in Fig. 1 represent locations and names in 2009.

### Fish Collections and Total Mercury Analysis

Using a dip net, a grab sample of 100–250 mosquitofish (*Gambusia* spp.) was collected at each monitoring station annually. Mosquitofish are indicators of short-term, localized changes in water quality because of their small range, short life span, and widespread occurrence in the Everglades. Adult mosquitofish typically forage on zooplankton, insects, and other invertebrates. After collection, the mosquitofish (entire bodies) were homogenized using a Polytron homogenizer and each aliquot was analyzed for THg. The final sample concentration was determined from the average of three to five aliquots. Up to 20 sunfish (*Lepomis* spp.) were collected annually using electroshocking techniques. Each whole fish was analyzed for THg. Sunfish are thought to have an average life span of 4–7 years in the wild. They are prevalent in the Everglades and are the preferred prey for a large number of fish-eating wildlife including wading birds; thus, sunfish are an indicator of mercury exposure. Over the 11-year period, several sunfish species were caught: warmouth (*L. gulosus*), spotted (*L. punctatus*), bluegill (*L. macrochirus*), and redear (*L. microlophus*). Even though mercury concentration can vary by species, we combined all sunfish data since interspecies THg variation was not an issue at this level of analysis. Adult sunfish diet consists of insects (various flies), snails, and crayfish. Also using electroshock methods, up to 20 LMB (*Micropterus salmoides*) were collected annually and the fillets were analyzed for THg. LMB are long-lived and are indicators of human exposure to mercury. Adult LMB diet includes various small fishes (e.g., bluegill), crayfish, frogs, baby alligators, and snails. Best efforts were made to collect 20 LMB and 20 sunfish annually from the designated sampling location(s); however, few fish were available for several years, therefore, quotas vary. In total, 1,993 LMB, 2,559 sunfish, and 484 mosquitofish aliquots were collected over the 11-year period (does not include QA/QC samples). Mercury concentration in each fish sample was determined using THg analysis. More than 85 % of the mercury found in the muscle tissue of fish is in methylated form (Grieb et al. 1990; Bloom 1992). Therefore, analyzing fish tissue for THg, a more straightforward and less costly procedure than analyzing for MeHg, can be interpreted as equivalent to the analysis of MeHg. Over the 11-year period, THg data for this program were analytically generated by means of SFWMD and the Florida Department of Environmental Protection (FDEP), both of which are certified by the Florida Department of Health under the National Environmental Laboratory Accreditation Program (NELAC). SFWMD used USEPA Draft Method 1631 (Mercury in Water by Oxidation, Purge and Trap, and Cold Vapor Atomic Fluorescence Spectrometry) for THg detection in fish tissue, and FDEP used USEPA Method 245.6 (Mercury in Tissues by Cold Vapor Atomic Absorption Spectrometry). Both methods apply performance-based standards and appropriate levels of QA/QC as required by NELAC. Recent records indicate no meaningful difference in fish tissue THg level between the two methods (Gabriel et al. 2011a).

### Surface Water Sulfate Collection and Analysis

On a quarterly basis, 125-mL filtered (0.45 μm) grab samples of surface water were collected at 0.5-m depth, or half the total water depth if the depth was less than 0.5 m, from all stations and analyzed for sulfate. A sample was not taken if there was not an adequate water depth. Sulfate in each sample was determined by SFWMD using an ion chromatographic method (USEPA method 300.0) with a detection limit of 0.1 mg/L for sulfate. A Dionex ICS 3000 ion chromatograph was used to measure sulfate. We chose surface water as a means to observe sulfate level since it is less labor-intensive to sample and more cost-effective to monitor and control compared to sediment pore water which will help expedite and simplify future assessment of fish THg levels throughout the EPA. In total, over the 11-year period, 2,360 sulfate samples were collected (does not include QA/QC samples).

### Field and Laboratory Quality Assurance/Quality Control (QA/QC)

SFWMD and FDEP employ stringent QA/QC programs for mercury collection and analysis due to its ultra-trace concentrations in the environment. The goals of both QA programs are to ensure that: (1) standard collection, processing, and analysis techniques are applied consistently and correctly; (2) the number of lost, damaged, and uncollected samples are minimized; (3) integrity of the data is maintained and documented from sample collection to record entry; and (4) data are usable based on project objectives. QC measures include internal and external sample checks. Concerning fish collections used for THg analysis, typical internal QC checks included replicate measurements/samples, internal test samples, method validation, blanks, and use of standard reference materials. Typical external QC checks for contracted laboratories included split samples (SS), blind studies, independent performance audits, and periodic proficiency examinations. For calendar year 2009, the mean relative standard deviation (RSD) between replicate and routine samples for the 42 obtained mosquitofish aliquots was 11.7 %. Three of the 14 RSDs were greater than the required 20 % QA/QC precision level indicating no relative concern. To review split and replicate sample data for previous years see South Florida Environmental Report (SFER), Chap. 3B-1 http://www.sfwmd.gov/sfer. Round-robin studies for fish mercury were also routinely initiated to ensure further reproducibility between mercury-sampling initiatives and to evaluate the performance of both laboratories. The most recent report summarizing the interlaboratory investigation can be obtained through http://www.dep.state.fl.us/labs/default.htm. To review surface water sulfate QC criteria see SFER, vol III, Appendix 3-2 at Xue et al. (2012) and vol 1, Chap. 3A at Payne and Xue (2012).

### Data Standardization

Interpretability of mercury levels in fish can be problematic due to confounding influences of age and species. To provide an unbiased view of fish THg/sulfate relationships and to reduce fish THg data variability, we standardized LMB and sunfish concentrations. Only age-1 LMB were used for analysis as this was the most abundant age from 1998 to 2009 across all stations. Using age-1 LMB also provides a conservative assessment of mercury exposure to humans and wildlife as mercury concentration increases with fish age. After filtering by age-1, 679 LMB of the 1,993 collected (see above) were available for data analysis. Sunfish concentration can also vary with age; however, SFWMD does not determine the age of sunfish after collection. Instead, we normalized (divided) all sunfish THg levels by fish length which is a suitable proxy for age. The average length for an adult sunfish in the Everglades is between 5 and 9 in. (127–228 mm).

## Results and Discussion

### Relationships Between Fish THg and Sulfate

**Fig. 3:**
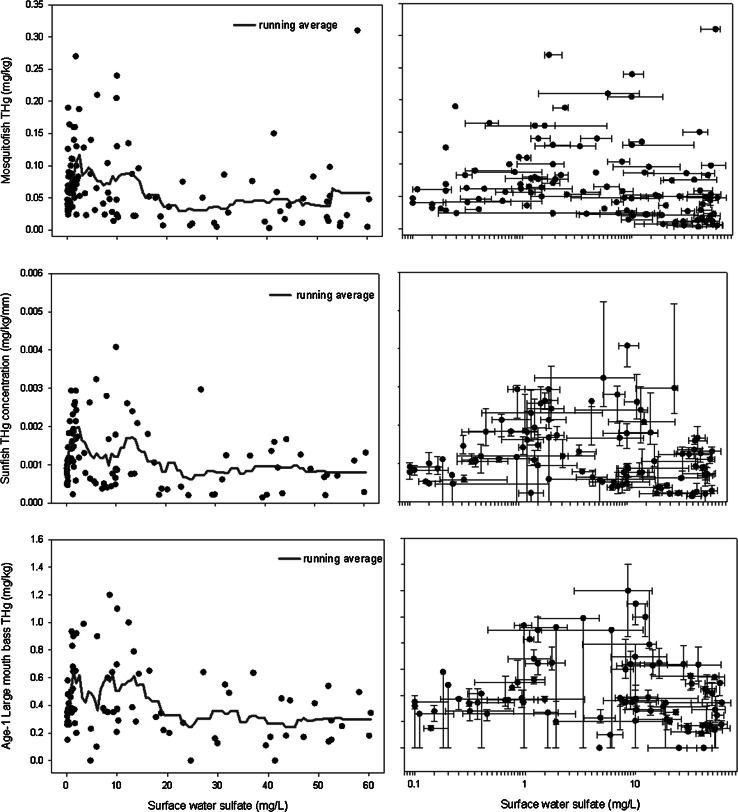
Fish THg and sulfate (SO_4_^2−^) relationships: the points in each plot display the median value for each station for each year. Plots on the right include the 25th and 75th percentiles and a log-transformed *x* axis. Data points in these plots were developed from 679 age-1 largemouth bass, 2,559 length-standardized sunfish, 484 mosquitofish aliquots, and 2,360 surface water sulfate samples from 12 fish stations and 12 sulfate stations over 11 years (1998–2009). Results for mosquitofish do not have percentile data because one averaged-based sample (aliquot) was obtained per year for each station. Not all stations contain the same number of data points for each year because of limitations in sample collection for specific years (e.g., not enough fish or adequate water depth)

The relationship between sulfate and THg in each fish type is nonlinear and resembles a skewed trend (Fig. 3). Fish THg in each type abruptly increases up to ~1 mg/L sulfate (Fig. 3), displays peak THg levels between 1 and 12 mg/L sulfate, has a downward sloping trend between 12 and 25 mg/L sulfate then a slight downward sloping to zero-slope trend for sulfate concentrations ≥25 mg/L. A report by Pollman (2012) shows the same trend for mosquitofish and LMB using Regional Environmental Monitoring Assessment Program (REMAP) data from 1996 to 2006. In that study, peak fish THg levels were present in surface waters with sulfate concentration between 1 and 10 mg/L. The fish THg and sulfate relationships in our study are highly similar to relationships between MeHg production and sulfate in sediment pore water observed in other studies (Gilmour et al. 1992, 2007; Benoit et al. 1999a, b). Field and laboratory studies show sulfate stimulates MeHg production under MSR, but as sulfate is reduced to sulfide, the sulfide can bind with Hg^2+^ which limits its availability for methylation (Gilmour et al. 1992, 2007; Benoit et al. 1999a, b). The reaction of sulfide with Hg^2+^ to produce insoluble cinnabar (HgS) has been described as a principal mechanism of decreasing mercury availability for methylation in aquatic systems (Gilmour et al. 1998; Orem et al. 2011). Sulfide may also inhibit MeHg production with the formation of sulfhydryl–Hg complexes in DOC that can restrict the bioavailability Hg^2+^ (Orem et al. 2011; Aiken et al. 2011). This dual effect of sulfur on methylation results in maximum MeHg production in so-called “Goldilocks” zones where sulfate and sulfide levels are just right for mercury methylation (Frederick et al. 2005). Mercury methylation rates in Everglades surface waters are generally the highest at 2–20 mg/L sulfate with moderate pore water sulfide concentrations (5–150 μg/L) (Gilmour et al. 2007). Sulfide begins to repress mercury methylation at concentrations above ~300 μg/L in pore water (Gilmour et al. 1992, 2007; Benoit et al. 1999a, b, 2003; Axelrad et al. 2008). While mercury methylation and MeHg bioaccumulation are two distinct biological processes, the plots in Fig. 3 show the connection between an important constituent for mercury methylation (sulfate) and an end product of Hg bioaccumulation (fish THg). Because fish THg concentrations (this study) and MeHg production (Gilmour et al. 1992, 2007; Benoit et al. 1999a, b) share peak levels of concentration/production between 1 and 20 mg/L sulfate, this suggests that an important driver for the observed trend between fish THg and sulfate in this study is MeHg production by MSR. All fish types show the same general trend in relation to sulfate; therefore, transfer rates of mercury to fish may be limited by MSR rather than by differences between fish types in their ability to bioaccumulate mercury. Each plot shows large variations in fish THg. This is caused by several factors affecting mercury methylation and fish mercury bioaccumulation. Primary factors affecting mercury methylation are spatiotemporal variation in quality and quantity of DOC (Cai et al. 1999; Benoit et al. 2001; Reddy and Aiken 2001; Drexel et al. 2002; Miller et al. 2007), bioavailable inorganic mercury (Benoit et al. 1999a, b; Kelly et al. 2003), redox conditions (Marvin-DiPasquale et al. 2003; Hollweg et al. 2009), concentration of other dissolved ions (Jay et al. 2002; Slowey and Brown 2007), and pH (Kelly et al. 2003). Primary factors affecting mercury bioaccumulation are water temperature (Bodaly et al. 1993; Ethier et al. 2008), fish feeding patterns (Li et al. 2009; Riva-Murry et al. 2011), growth rates (Simoneau et al. 2005), and migration (Eagles-Smith et al. 2008; Baker et al. 2009). We attribute the variation in sulfate to changes in station location and wetland biogeochemistry which affects concentrations of sulfate and sulfide. Considering the large number of factors that can influence mercury methylation and bioaccumulation and sulfate concentration, it is quite surprising to observe any relationship between surface water sulfate and fish THg. An informative next step would be to quantify these THg/sulfate relationships for lower food chain organisms (e.g., periphyton, insects, and zooplankton) to determine whether trends change with organism order or trophic level.

For each of the three fish species, there is a substantial number of high-fish THg observations (e.g., 20 % of total for LMB) in high-sulfate levels (>30 mg/L). In this study, we identify high-fish THg as >0.4 mg/kg for age-1 LMB, >0.05 mg/kg for mosquitofish and >0.001 mg/kg/mm for sunfish. The presence of high-fish THg levels in high-sulfate conditions (Fig. 3) complicates the interpretation of an “optimum” sulfate/sulfide concentration for mercury methylation as noted by other researchers (Gilmour et al. 1992, 2007; Benoit et al. 1999a, b, 2003; Frederick et al. 2005; Axelrad et al. 2008). Some potential biogeochemical-related justifications for the high-fish THg levels in high-sulfate conditions are as follows: (1) Intense rainfall produced by convective air masses systems, particularly during warmer periods of the year, efficiently scavenges atmospheric Hg^2+^ (Guentzel et al. 2001; Seo et al. 2011), which deposits “pulses” of bioavailable Hg^2+^, and these pulses may produce short-term enhancements in MeHg production in surficial sediment; (2) In locations/instances where high sulfate is present, there may also be elevated organic substrate from sediment disturbance and resuspension which could enhance Hg^2+^ mobility (Ravichandran et al. 1998; Golding et al. 2002) and the quantity of electron donors (e.g., acetate, lactate, propionate) for methylation (Achá et al. 2011); (3) In high-sulfate/sulfide conditions, specific bioavailable charged (Li et al. 2010; Golding et al. 2008; Kelly et al. 2003) and uncharged (Benoit et al. 1999a, b; Jay et al. 2002) mercury-sulfide species may be in excess and thus enhance methylation (Benoit et al. 1999a, b; Kelly et al. 2003; Li et al 2010). For example, Jay et al. (2002) found through model simulation that the formation of polysulfides (e.g., Hg(SH)_2_^0^, HgS_2_H^−^, HgSH^+^) in natural waters may decrease methylation rates, except when cinnabar is present. In the absence of polysulfides, Hg_(aq)_^0^ is the dominant species at low sulfide. At higher levels, HgS_2_H^−^ becomes the dominant complex resulting in a decrease in Hg_(aq)_^0^ and a subsequent decrease in methylation; (4) Methylation of mercury can occur in wetland compartments (e.g., periphyton biofilms) that may be less affected by high-sulfate levels (Achá et al. 2011, 2012; Correia et al. 2012); (5) Areas that contain a high concentration of sediment THg (Cohen et al. 2009) may provide an excess source of mercury for methylation to counterbalance a high-sulfide condition. (6) Anomalous fish migration patterns (e.g., a larger than typical migration radius that covers areas with low and high methylation or bioaccumulation) and feeding patterns (e.g., feeding only from higher food chain organisms).

### Ecosystem Management Implications

Figure 3 shows that health-concerning fish THg levels to humans^1^ and wildlife^2^ may occur in nearly all surface water sulfate conditions throughout the EPA. Twenty-seven percent of all mosquitofish THg aliquots were above USEPA’s trophic level 3 criterion, 11.6 % of all sunfish THg samples were above the trophic level 4 criterion, and 10.1 % of all LMB THg samples were above FDEP’s “No consumption” criterion for children and childbearing age women. Further complicating the issue, wetland biogeochemistry and MeHg production in the EPA can display extreme spatiotemporal variability (Rumbold and Fink 2006; Scheidt and Kalla 2007; Pollman 2012). Gabriel et al. (2011b) note certain areas continue to be MeHg “hot spot” areas; however, other areas are showing reverse THg trends, such as LMB THg increases at sites HOLYBC in the Holey Land Wildlife Management Area and WCA2U3 in Water Conservation Area-2. Site L67F1 in the Everglades National Park, which has shown the highest fish THg levels since the beginning of the period of record (early 1990s), has shifted toward lower concentrations, particularly for large-bodied fish. The commonly observed north-to-south spatial trend in fish THg is changing, with concentrations becoming more uniform across the lower to middle portion of the EPA. The ever-changing signature of MeHg production (Gabriel et al. 2011b; Scheidt and Kalla 2007), mixed with the complex combination of conditions that produce MeHg creates moving targets and makes it impractical, at least over the long term, to focus on transient hot spot areas as a means of managing MeHg production.

To decrease the risk of exposure to toxic MeHg, factors promoting methylation must be addressed. Reduction in wetland area is obviously not consistent with Everglades restoration goals, although minimizing the occurrence of dry/rewet cycles, where possible, could decrease spikes in MeHg production. Reducing DOC could impede MeHg production, but reducing DOC is not realistic in a peat-forming environment. Because a large percentage of mercury from atmospheric deposition is from long-range atmospheric transport originating outside the United States (Axelrad et al. 2008; Gu et al. 2012), further reductions in atmospheric input of mercury to the Everglades would require international cooperation. This leaves control of sulfate inputs as the most feasible option for reducing MeHg production and bioaccumulation in the Everglades. The Comprehensive Everglades Restoration Plan (CERP) has a performance measure of 1 mg/L sulfate for the Everglades surface waters (RECOVER 2011). The data in this study show health-concerning fish THg levels can be present in all sulfate conditions (Fig. 3); however, there are few instances of excessively high-fish THg levels [≥0.6 mg/kg (age-1 LMB), ≥0.1 mg/kg (mosquitofish), ≥0.002 mg/kg/mm (sunfish)] in water with <1 mg/L or >20 mg/L sulfate. Only 5 % of all LMB THg samples were above 0.6 mg/kg in the <1 mg/L and >20 mg/L sulfate ranges. There were similar percentages for mosquitofish and sunfish THg levels in these sulfate ranges. Fish THg levels decline to acceptable or nonproblematic levels as sulfate levels approach zero sulfate. Therefore, while the 1 mg/L sulfate CERP performance measure is a highly ambitious goal, decreasing ambient surface water sulfate to 1 mg/L would significantly reduce MeHg risk which is consistent with the recommendations provided by Orem et al. (2011) and Corrales et al. (2011). Alternatively, as this study suggests, maintaining >20 mg/L sulfate conditions may also significantly reduce MeHg risk.

Management challenges with implementing the CERP 1 mg/L measure, or one similar, are as follows: (1) The Everglades is underlain by ground water that is higher in sulfate [20–58 mg/L (Bates et al. 2002)] and surrounded by seawater [~2,700 mg/L (28.93 mM) (Pilson 1998)] that can interact with the fresh water Everglades through atmospheric deposition, seepage, tidal effects, and surface water-groundwater interaction; (2) The Everglades receives continuous drainage from Everglades Agricultural Area (EAA) soils that contain sulfur from legacy applications and natural processes (Schueneman 2001; Ye et al. 2010; Orem et al. 2011) and drainage from STAs that contain sediments which at times and locations have high-oxidized sulfur levels; and (3) Approximately 25 % of the water entering the Everglades originates from Lake Okeechobee (30–40 mg/L surface water sulfate) by way of canal delivery (James and McCormick 2012). Despite the surrounding influence of groundwater and atmospheric deposition, current evidence shows neither are major sulfur sources to the Everglades system (Orem et al. 2011; James and McCormick 2012), at least currently. Therefore, the most direct method for altering ecosystem sulfate levels is through management of water quality and quantity discharges from the EAA, STAs, and Lake Okeechobee. Whether feasible or not from a water management perspective, altering ecosystem surface water sulfate levels would have a significant impact on mercury methylation, particularly if levels were dropped to 1 mg/L or maintained at levels >20 mg/L (Fig. 3). Most freshwater wetland areas in the lower EPA have sulfate ranging from <0.1 to 1 mg/L. Levels >60 mg/L can be found in the northern Everglades near canals outlets (Bates et al. 2002; Orem et al. 2011). Orem et al. (2011) and Corrales et al. (2011) provide detailed information on sulfur sources in EPA and offer potential management strategies to achieve reduced sulfur levels.

## Abbreviations

CERP: Comprehensive Everglades Restoration Program
EPA: Everglades Protection Area
MSR: Microbial sulfate reduction
THg: Total mercury
MeHg: Methyl-mercury
LMB: Largemouth bass
WCA: Water Conservation Area
DOC: Dissolved organic carbon
SFWMD: South Florida Water Management District
USEPA: U.S. Environmental Protection Agency
FDEP: Florida Department of Environmental Protection
QA/QC: Quality assurance/quality control
SFER: South Florida Environmental Report
SS: Split sample
RSD: Relative standard deviation
NELAC: National Environmental Laboratory Accreditation Program
REMAP: Regional Environmental Monitoring Assessment Program

## Footnotes

1 E.g., Florida Department of Health (FDOH) fish criterion for “No Consumption” by the general population (1.5 mg/kg), and “No consumption” by women of childbearing age and children (0.85 mg/kg) (FDOH 2008).

2 E.g., U.S. Fish and Wildlife Service (USFWS) predator protection criterion (0.100 mg/kg) (Eisler 1987), USEPA criteria of 0.077 and 0.346 mg/kg for trophic level 3 and 4 fish (USEPA 1997).
